# Detection of meningovascular neurotuberculosis through intracranial vessel wall imaging

**DOI:** 10.1590/0037-8682-0434-2021

**Published:** 2021-11-12

**Authors:** Paulo Márcio Borges Daniel, Flávia Sprenger, Bernardo Corrêa de Almeida Teixeira

**Affiliations:** 1 Universidade Federal do Paraná, Hospital de Clínicas, Departamento de Radiologia, Curitiba, PR, Brasil.

**FIGURE 1:**
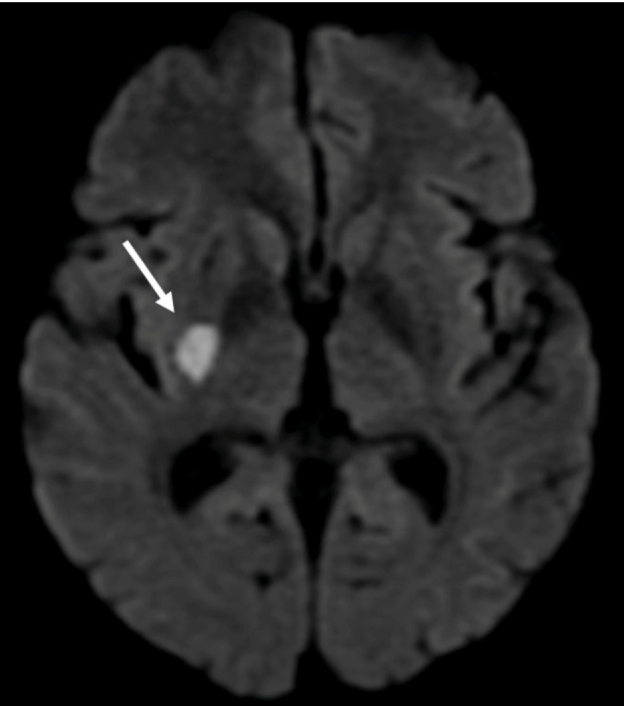
Axial diffusion-weighted image showing acute infarction in the right lentiform nucleus (white arrow).

**FIGURE 2:**
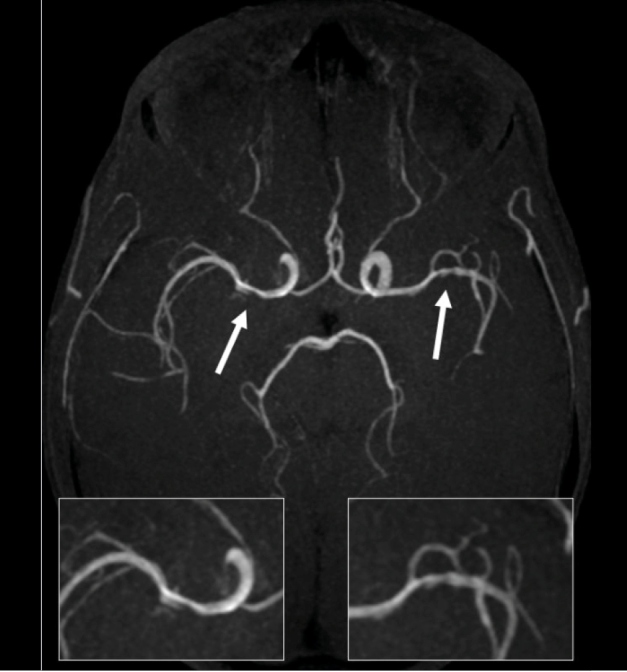
Time-of-flight angio-MRI sequence demonstrating irregular stenosis (white arrows) along medium cerebral arteries.

**FIGURE 3:**
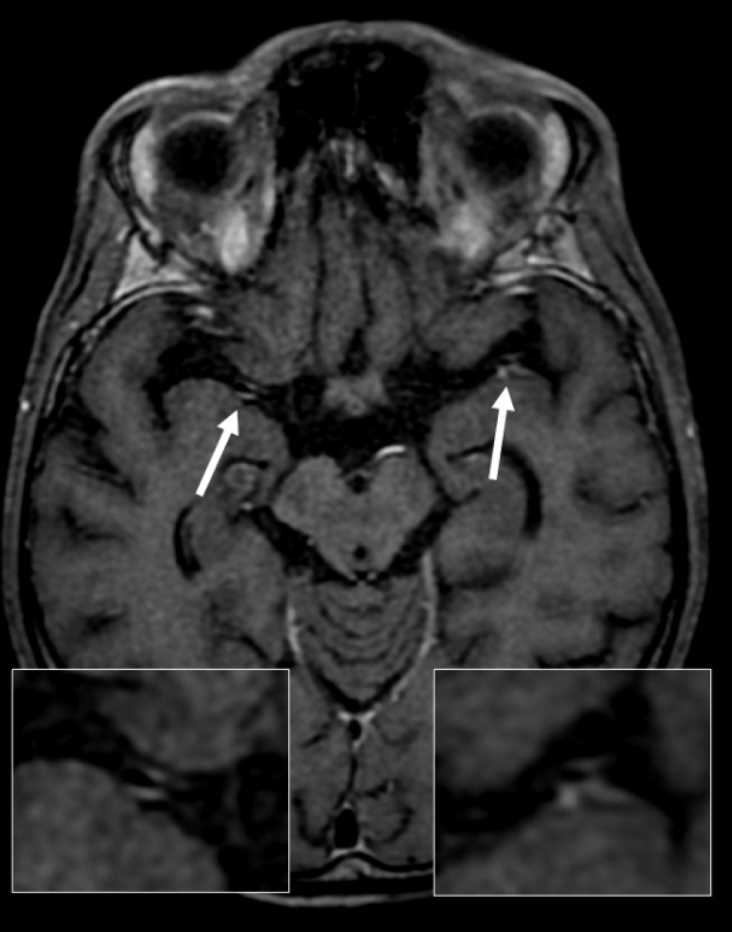
Vessel wall imaging sequence demonstrating circumferential parietal contrast enhancement (white arrows) in the right M1 and left M2 segments.

A 35-year-old homeless man presented with a sudden loss of left limb strength with brachial predominance and a recent history of weight loss and cough. Brain magnetic resonance imaging (MRI) revealed patchy foci of leptomeningeal nodular enhancement and acute ischemia in the right lentiform nucleus (Figure 1). Vessel wall imaging revealed circumferential smooth enhancement in the right M1 and left M2 segments, suggestive of vasculitis secondary to an inflammatory/infectious process (Figures 2 and 3).

The patient was HIV positive, with immunosuppression stigmas, such as oropharyngeal candidiasis, and off antiretroviral therapy (CD4: 9 cells/mm³ and viral load: 364.891/mL). Chest computed tomography revealed diffuse miliary lung nodules; a cerebrospinal fluid polymerase chain reaction test was positive for neurotuberculosis.

Cerebral vasculitis, which corresponds to the inflammation of blood vessel walls, may occur as a complication of neurotuberculosis and lead to secondary strokes^1^ ^,^ ^2^ ^,^ ^3^.

Magnetic resonance vessel wall imaging is a non-invasive method capable of detecting vascular inflammation, and arterial abnormalities may persist after treatment. Moreover, residual vascular lesions may be a source of disease recurrence. Therefore, follow-up studies may be helpful^1^ ^,^ ^2^.

Vessel wall enhancement with the absence of vascular complications, such as infarction, may significantly improve prognosis, with early treatment initiated to prevent complications^1^ ^,^ ^2^.
